# Substantial Insect Herbivory in a South African Savanna‐Forest Mosaic: A Neglected Topic

**DOI:** 10.1002/ece3.70466

**Published:** 2024-11-09

**Authors:** Heveakore Maraia, Tristan Charles‐Dominique, Kyle W. Tomlinson, Ann Carla Staver, Leonardo Re Jorge, Uriel Gélin, Jitka Jancuchova‐Laskova, Legi Sam, Dawood Hattas, Inga Freiberga, Katerina Sam

**Affiliations:** ^1^ Institute of Entomology, Biology Centre Czech Academy Sciences České Budějovice Czech Republic; ^2^ Faculty of Science University of South Bohemia České Budějovice Czech Republic; ^3^ CIRAD, CNRS, INRAE, IRD, UMR AMAP University of Montpellier Montpellier France; ^4^ CNRS UMR7618, Institute of Ecology and Environmental Sciences Paris Sorbonne University Paris France; ^5^ Center for Integrative Conservation & Key Laboratory for Conservation of Tropical Rainforests and Asian Elephants, Xishuangbanna Tropical Botanical Garden Chinese Academy of Sciences Mengla Yunnan China; ^6^ Ecology and Evolutionary Biology and the Yale Institute for Biospheric Studies New Haven Connecticut USA; ^7^ Section of EcoInformatics and Biodiversity, Department of Biology, Centre for Biodiversity Dynamics in a Changing World (BIOCHANGE)5 Aarhus University Aarhus Denmark; ^8^ Section of EcoInformatics and Biodiversity, Department of Biology, Centre for Ecological Dynamics in a Novel Biosphere (ECONOVO) Aarhus University Aarhus Denmark; ^9^ Department of Biological Sciences University of Cape Town Cape Town South Africa

**Keywords:** arthropod herbivory damage, insect herbivory, insect–plant interactions, leaf chewers, leaf miners, savanna‐forest mosaic, South Africa, ungulates

## Abstract

Insect herbivory plays a crucial role in shaping plant communities in many terrestrial ecosystems. However, in African savannas, insect herbivory has been relatively understudied compared to large mammalian herbivory. In this study, we examined the impact of insect herbivory, focusing on leaf chewers and miners, in a South African savanna‐forest mosaic (including patches of forest, thicket and savanna) in Hluhluwe iMfolozi Park, South Africa. Our investigation spanned gradients of rainfall, fire frequency and mammal density. We surveyed a total of 864 woody plants from 48 plant species in 38 plots. Insects consumed 6% of leaf biomass, which is comparable to their impact in temperate broadleaf forests, but the extent of herbivory damage varied between vegetation types. Overall, leaf loss was 70% higher in forests and savanna than that in thicket. Plants in the forests experienced greater damage from chewing insects, whereas miners caused relatively more damage in savannas. Rates of insect herbivory also varied among plant species, declining with carbon and dry matter content but increasing with specific leaf area. Although no significant trade‐off was detected between insect and mammal herbivory, plant species with limited physical defences against mammals tended to experience high levels of insect herbivory. Our findings highlight the intricate dynamics of insect herbivory in different vegetation types and suggest that insect leaf herbivory, alongside mammalian herbivory, could play a significant role in influencing plant community composition and overall savanna ecosystem functioning.

## Graphical abstract

Our work represents a rare study of insect herbivory across a large national park, the Hluhluwe‐iMfolozi Park (900 km^2^) in South Africa. This study not only connects insect herbivory to key and typical savanna drivers: rainfall, abundances of mammals and fire frequencies, but also examines the impact of two insect feeding guilds (chewers and miners). Furthermore, the study correlates total herbivory and herbivory by feeding guilds to three distinct biomes in savanna‐forest mosaic, individual woody plant species, and their respective traits.

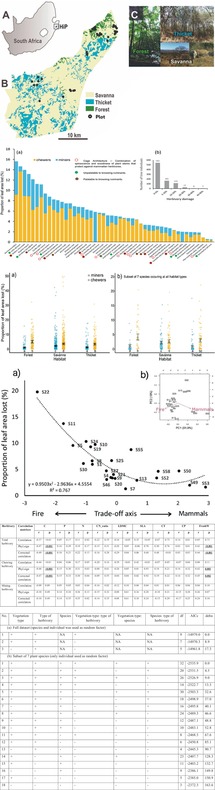

## 1 Introduction

In terrestrial ecosystems, insect herbivores play a significant role in shaping plant communities by consuming ~18% of plant biomass annually (Cyr and Pace 1993). Herbivorous insects cause varying levels of leaf loss across biomes: ~7.1%, ~10% (Coley and Barone 1996) and 50%, respectively, in temperate forest, tropical forest and Neotropical savannas (Lopes and Vasconcelos 2011). However, insect herbivory remains understudied in many regions and ecosystems worldwide (Liu et al. 2024), particularly in areas with diverse communities of large herbivorous mammals known to have strong impact on vegetation (Charles‐Dominique et al. 2016). Consequently, there are no studies that disentangle the relative importance and the consequences of simultaneous herbivory by insects versus mammals on plants (Hambäck and Beckerman 2003).

In African savannas, where large mammal communities have remained almost intact (Charles‐Dominique et al. 2016), water availability and fire are described as the main environmental drivers of vegetation structure, biodiversity, distribution and traits (Lehmann et al. 2011; Staver, Archibald, and Levin 2011). These drivers have cascading effects on other organisms, including insects. Insect herbivory plays a central role on plant biomass and ecology in many parts of the world (Hambäck and Beckerman 2003), but its impact on savanna vegetation has been largely overlooked (Davies et al. 2016; Goheen and Palmer 2010; but see for example Sinclair 1975). The ecological importance of insect herbivory depends on both internal and external factors influencing their diet (Forister et al. 2015), including feeding guild (Novotny et al. 2010), nutritional requirements (Wilson et al. 2019) and plant defences (War et al. 2012).

Herbivorous insects are classified into several feeding guilds based on their feeding behaviour and the plant parts they consume (Novotny et al. 2010). Leaf chewers, such as caterpillars and grasshoppers, are often generalists, more mobile and consume the external parts of leaves (Adu‐Acheampong and Samways 2019; Hughes et al. 2015). Leaf miners, usually larvae, are highly specialised, feeding within the leaf tissue and creating visible tunnels or mines (Lopez‐Vaamonde, Kirichenko, and Ohshima 2021). A smaller proportion of herbivorous insects belong to other guilds such as sap suckers, gall makers, stem borers, root feeders and senescence feeders, which primarily target agricultural crops, especially in Africa (Abate and Ampofo 1996). Nonetheless, some African trees and shrubs, such as *Vachellia* and *Senegalia* species, are primarily attacked by beetles that feed on the stems and reproductive parts, such as seeds (Miller 1996; Mucunguzi 1995), which can significantly contribute to tree mortality. Considering these guilds helps in understanding the ecological roles of different insect species, their interactions with plants and the nutriments they access.

Herbivorous insects generally target plants with high leaf nitrogen (N) and phosphorus (P) as these nutrients promote their growth (Gu et al. 2022; Rode, Lemoine, and Smith 2017). Conversely, they avoid plants with high polyphenol content (e.g., condensed tannins) (Singh, Kaur, and Kariyat 2021; Singh and Kariyat 2020) and high leaf dry matter content (LDMC) (Roeder et al. 2022), as these factors decrease intake and digestibility due to their impact on leaf toughness, which is linked to high cellulose or lignin concentrations (Kitajima et al. 2012). Mammals, fire and climatic factors can influence insect herbivory partly through their effects on these compounds.

Herbivory by mammals may negatively impact insect herbivory by directly depleting leaf resources but inducing chemical response of plants to herbivory such as higher concentration of phenolics (DuToit, Bryant, and Frisby 1990; Singh, Kaur, and Kariyat 2021; War et al. 2012). Conversely, mammal herbivory can indirectly favour insect herbivory by stimulating the growth of new leaves that are less defended, at least during their initial development but also through cascading effects. For instance, investment in structural defence against mammals by woody plant species, such as spines and cages (Charles‐Dominique et al. 2017; Shipley 2007), is expected to reduce resources available for alternative carbon‐based defences (cellulose, lignin, polyphenols) and be linked to greater leaf nitrogen content (Hanley et al. 2007; Tomlinson et al. 2016; Wigley, Fritz, and Coetsee 2018; Wigley et al. 2019). Altogether, this suggests that plants with structural defences may be more nutritious, less chemically defended due to energy investment in structural defence and therefore more attractive to insect herbivores (Arnone et al. 1995; Kimmerer and MacDonald 1987; Wilson et al. 2019).

Environmental factors such as water availability, temperature and fire can affect plants chemistry and insect herbivory notably through their impact on nutriment allocation (Jaworski and Hilszczański 2013; Kuczyk, Müller, and Fischer 2021). Trade‐offs are expected in resource‐limited environments, such as arid or seasonally dry ecosystems (Tomlinson et al. 2016). LDMC, N and insect abundance are expected to decrease in drier environment or season. Fire may have similar impact than herbivorous mammals on insect herbivory by removing dead leaves from plants, promoting a rapid flush of new leaves and providing a source of less defended material (Lopes and Vasconcelos 2011; Radho‐Toly, Majer, and Yates 2001). Whether frequent fire enhances or reduces leaf nutritional quality to insects is unclear (Rieske, Housman, and Arthur 2002).

Considering these factors together is crucial to disentangle the relative importance of each factor on insect herbivory, which may also be specific to feeding guild. For instance, increased leaf toughness induced by drought (but possibly reduced by adaptation to mammal herbivory), should more strongly affect leaf chewers (‘chewers’) (Gely, Laurance, and Stork 2020; Louda and Rodman 1996; Price 1991).

In this study, we tested how insect herbivory varies with leaf nutrient traits of host tree species, mammal herbivore density, fire frequency and the interactions between these factors in an African savanna‐forest mosaic composed of 3 vegetation (or habitat) types (savanna, thicket and forest) interspersed with one another in Hluhluwe‐iMfolozi Park (HiP), South Africa. We hypothesised that savannas would experience lower insect herbivory than forests and thickets due to greater competition for food resources with large mammals in savannas. However, within savanna plots, we further anticipated that insect herbivory would increase with mammal herbivory density, fire frequency and rainfall due to regrowth and flush of new leaves after these disturbances.

## 2 Materials And Methods

### 2.1 Study Site

**FIGURE 1:**
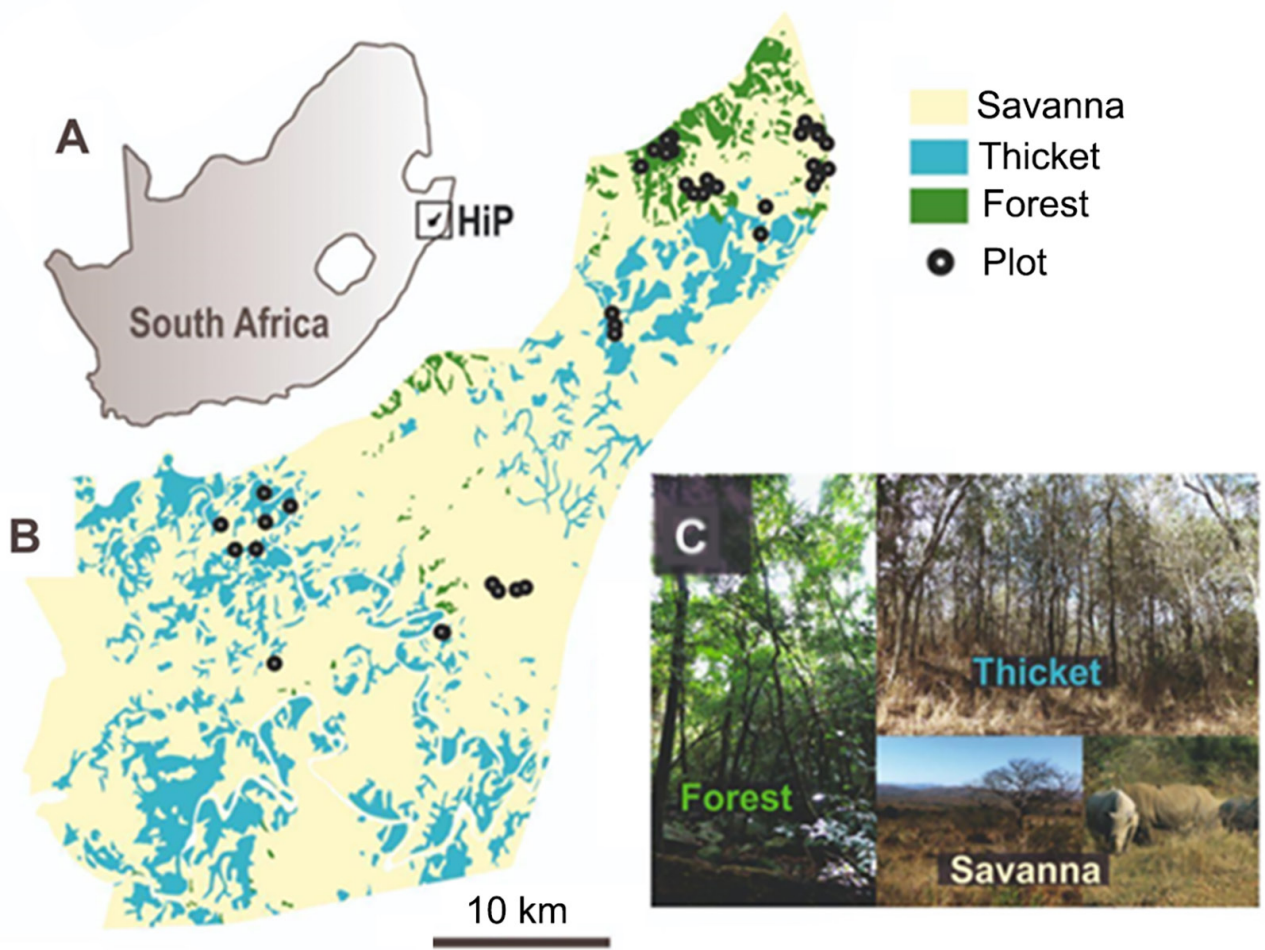
Location of Hluhluwe‐iMfolozi Park within South Africa (A), vegetation type distribution in HiP with location of 38 study plots (B) and photographs showing intact forest, thicket and savanna (C).

Hluhluwe‐iMfolozi Park is located in northern KwaZulu Natal, South Africa (Figure 1). The park covers an area of 900 km^2^ (28°000 S to 28°430 S, 31°700 E to 32°140 E) and has a varied topography, with hills and valleys ranging from 40 to 750 metres (m) above sea level (Wigley et al. 2015). The park's vegetation has been categorised into three main vegetation types: (1) savanna with discontinuous tree cover and a continuous layer of grasses, (2) thicket with dense shrub, a variable understory, and a 4 to 6 m tall open canopy and (3) forest, described as tall (> 10 m) woody vegetation with no grass layer and no intermediate shade‐tolerant tree layer (Charles‐Dominique, Staver, et al. 2015). Each of the 38 study plots used in this study had previously been classified into one of these three types of vegetation types (Charles‐Dominique, Staver, et al. 2015; Table S1). The diversity of soil types in the area is a result of variations in topography, geology and climate (Boundja and Midgley 2010). Soil texture (e.g., clay and sand) also plays important roles in determining the relationship between tree cover and rainfall patterns in African savannas (Case and Staver 2017, 2018) and also influences nutrient availability. The dominant soils consist of shales and sandstones, with dolerite, tillite, granite and basalt interspersed (Whateley and Porter 1983). Rainfall patterns are associated with elevation (Balfour and Howison 2002), with higher elevations receiving a mean annual rainfall of 975–1000 mm, while lower elevations receive < 600 mm. Rainfall mainly occurs during the summer months of October to March (Balfour and Howison 2002). The park is home to a variety of large mammal herbivores including elephant (*Loxodonta africana*), black and white rhinoceros (*Diceros bicornis* and *Ceratotherium simum*, respectively), wildebeest (*Connochaetes taurinus*), plains zebra (*Equus quagga*), giraffe (*Giraffa camelopardalis*), cape buffalo (*Syncerus caffer*), nyala (*Tragelaphus angasii*), impala (*Aepyceros melampus*) and grey duiker (*Sylvicapra grimmia*) (Charles‐Dominique, Staver, et al. 2015; Staver et al. 2009). Two species are more associated with forest, namely red duiker (*Cephalophus natalensis*) and cape bushbuck (*Tragelaphus sylvaticus*). Elephant and buffalo also prefer forest but not in large numbers, compared to red duiker and cape bushbuck (Charles‐Dominique, Staver, et al. 2015).

### 2.2 Measurements of Insect Herbivory

All leaf material for insect herbivory measurement was collected in November 2017 (late spring). Using a stratified sampling method, we selected 38 plots across HiP, each of 40 × 10 m, representing three vegetation types (savanna, forest and thicket) with varying levels of fire frequency and mammal herbivory. Twenty‐four savanna plots, seven forest plots and seven thicket plots were selected. We first identified a list of 48 dominant woody species (Table S2), i.e. those that accounted for over 70% of the woody basal area in the park (Charles‐Dominique, Midgley, and Bond 2015). At each plot, we then searched for these focal species and sampled up to three individuals of each of them (if present), totalling to 864 individuals sampled across the 38 plots. We collected lateral branches containing 50–100 leaves per woody plant at breast height (1.3 m). The whole branches were labelled and transported in A3‐sized zip‐lock bags with moist tissue to the laboratory within 6 h. Individual leaves were separated from branches and scanned individually using an A4 scanner (CanoScan Lide 200, 600 dpi resolution). For species with compound leaves, each pinnule (leaflet) was considered as a single leaf. Each scan of these species consisted of several pinnules and several complete compound leaves. We sampled young and fully developed (mature) leaves on each branch. Mature leaves were scanned on both faces: 2/3 were scanned on their adaxial side (No. of scans = 17,009), and 1/3 on their abaxial side (No. of scans = 4618). Most of the woody plant species that we sampled had fewer young leaves with very low or no herbivory from the abaxial side by insect herbivores (chewers and miners), therefore, we only scanned their adaxial side (No. of scans = 4617). The evaluation of surfaces damaged by insect herbivores followed the protocol established by Sam et al. (2020). We outlined the absent leaf edges using the anticipated shape, converted the photographs to black and white in Photoshop software and computed the consumed area as well as the remaining leaf area in ImageJ software.

The herbivory estimation was performed in two steps, distinguishing herbivory damage caused by chewers and miners. This involved determining the extrapolated area of the complete leaf (i), the area consumed by chewers (ii) and the area damaged by miners (iii). In the case of miners, we measured the leaf‐miner tracks, specifically the leaf area consumed or damaged by the miners. Although miners do not entirely remove the plant tissue and only consume a portion of the leaf layer, this approach is widely employed (Basset 1991). For *Vachellia* spp., *Senegalia* spp. and *Dichrostachys cinerea*, herbivory was assessed on a subset of 30 pinnules (leaflets) and three complete leaves, considering the area of missing leaflets. Proportional herbivory damage was calculated by dividing the damaged leaf area (in cm^2^) caused by (i) chewers, (ii) miners or (iii) a combination of both (i + ii) by the expected leaf area (in cm^2^). There were no observed differences in damage between young and old leaves or between the abaxial and adaxial sides, so all values were averaged together (Figure S1). If only one side of a leaf was damaged, it was treated as herbivory impacting the entire leaf. It should be noted that this approach potentially overestimates the leaf damage caused by miners. Cumulative damage per scanned leaves of individual plants was used to calculate standardised leaf damage, expressed as the proportion of area lost (in cm^2^ /100 cm^2^ of foliage) for individual trees. This standardisation allowed for comparisons, as leaf area varied significantly among plant species and surveyed vegetation types due to habitat‐specific leaf size differences (Figures S2 and S3). Individual trees were used as sampling unit in the plot level and vegetation type level analyses. However, proportions of area lost were averaged across individuals of each plant species for species‐level analysis, as the traits to which we related the herbivory damage were collected at the species level from different individuals than we used in our study and unspecified plot from within the region (Charles‐Dominique, Beckett, et al. 2015; Charles‐Dominique, Staver, et al. 2015; Charles‐Dominique, Midgley, and Bond 2015). Overall leaf area loss from each individual plant was not calculable because we did not measure the total leaf area, or number of leaves, of each individual woody plant.

For plot‐level analysis, we determined the herbivory damage in biome‐specific units of cm^2^/100 cm^2^ of foliage caused by chewers, miners and overall herbivory. These values were then multiplied by the abundance of each specific plant species within the plot. In essence, the analysis focused on the vegetation level, calculating the average herbivory caused by chewing and mining for each species separately in the forest, thicket and savanna. To estimate vegetation type‐level herbivory damage, we first assessed plant species abundances in the field from the plot data and multiplied the abundances by herbivory damage identified from the collected leaf samples from individual plant species that occurred within the three vegetation types studied.

### 2.3 Explanatory Variables and Plant Traits

The leaf material for chemical analyses was collected as part of an earlier study in November 2013 (i.e., late spring, in the same month but in different year as leaf herbivory measurements) at the species level averaged across vegetation types and from different individuals than used in the current analysis of the herbivory. Therefore, the values were not vegetation type or plot specific, and we were able to work only at the level of plant species. For each of the 48 tree species, we randomly collected and pooled 20 g of mature and intact leaves from five different individuals aiming to have all three vegetation types represented by 1–2 individuals. All leaf samples were air‐dried and ground to pass through a 1 mm sieve. Milled leaf samples were sent to the Plant Sciences Laboratory, Department of Agriculture Western Cape (Elsenburg, Stellenbosch, South Africa) for the analysis of phosphorus (P), nitrogen (N) and carbon (C) concentrations using standard methods (Jones Jr, Wolf, and Mills 1991). Leaf condensed tannin (CT) concentrations were analysed using Sorghum tannin as a standard (Hattas and Julkunen‐Tiitto 2012). Crude protein (CP) was determined by the Kjeldahl procedure for nitrogen (Cooper, Owen‐Smith, and Bryant 1988). Leaf dry matter content (LDMC) was calculated as dry leaf mass (mg) divided by fresh weight (g). Specific leaf area (SLA) was calculated as leaf surface area (mm^2^) divided by leaf dry mass (mg) (Cornelissen et al. 2003). Information regarding the presence or absence of cage architecture formed by thorns and branches for each plant species was taken from Charles‐Dominique et al. (2017). We quantified the structural defence effectiveness of each plant species against herbivorous mammals by measuring the amount of phytomass (in grams per bite) that a herbivore could remove in each bite using the Bite Size Index (BSI), i.e. a bite size of a mammalian herbivore. BSI was calculated as the mean fresh weight of 10 human bites taken on five different plants of the same species (Charles‐Dominique, Midgley, and Bond 2015; Wigley et al. 2014). Palatability classification of woody plants to browsing ruminants (goats, impalas and kudus) was obtained from Owen‐Smith and Cooper (1987) and Charles‐Dominique et al. (2017) for a subset of the tree species (Table S3). With respect to environmental drivers, dung counts, which we obtained for each plot at the time when the survey of herbivory was conducted, were employed for assessing relative mammal herbivore activity (following methodology by Cromsigt et al. 2009). Fire count data for each plot for the period 1992–2012 was taken from Charles‐Dominique, Beckett, et al. (2015) (Table S1). Mean annual rainfall (MAR) data was derived from a rainfall map provided by the dung beetle research station.

### 2.4 Statistical Analyses

We evaluated standing insect herbivory at several levels (plant species irrespective to vegetation type, vegetation type, and plot). Standing herbivory is defined as the herbivory present on a foliage at a given time, and thus differs from herbivory rate, which is defined as a change in herbivory damage over a unit of time. *Plant species level*: First, we tested whether total, chewer, and/or miner herbivory at plant species level was correlated (via Pearson correlations) with any of the measured plant leaf traits (nutrients, chemical defences, physical traits). We calculated the mean insect herbivory of each type per woody plant species irrespective of their plot. We generated a phylogenetic tree of 48 plant species using the ‘V.PhyloMaker’ package in R (Jin and Qian 2019), then used the ‘phytools’ package (Revell 2012) to calculate phylogenetically weighted regressions between the different types of insect herbivory and plant species traits (Table 1). We also tested whether the different types of insect herbivory were correlated with defences against mammals, including caginess, palatability and bite size index (see descriptions above).

**TABLE 1:** Correlations (Pearson correlation) between the types of herbivory (total, chewing and mining) and plant traits, phylogenetic signal in the given correlation (phyl. sign) and corrected correlation between the types of herbivory and plant traits.

Herbivory	Correlation metrices	C		P		N		CN_ratio		LDMC		SLA		CT		CP		FreshW	
		*r*	*p*	*r*	*p*	*r*	*p*	*r*	*p*	*r*	*p*	*r*	*p*	*r*	*p*	*r*	*p*	*r*	*p*
Total herbivory	Correlation	−0.37	< 0.01	0.05	0.17	0.11	0.02	−0.22	0.35	−0.34	< 0.05	0.33	< 0.05	−0.07	0.75	0.14	0.01	0.05	0.73
	Phyl.sign	−0.47	**< 0.001**	0.16	0.29	0.19	0.17	−0.15	0.31	−0.28	0.05	−0.06	0.70	0.30	0.76	0.03	0.81	0.42	**< 0.001**
	Corrected correlation	−0.49	**< 0.001**	0.18	0.21	0.22	0.13	−0.16	0.28	−0.29	0.04	0.06	0.68	0.48	< 0.01	0.03	0.83	0.44	**< 0.001**
Chewing herbivory	Correlation	−0.44	< 0.01	0.04	0.06	0.17	0.05	−0.20	0.16	−0.39	< 0.01	0.42	< 0.01	−0.07	0.05	−0.07	0.04	0.08	0.11
	Phyl.sign	−0.48	**< 0.001**	0.15	0.30	0.31	0.03	−0.08	0.61	−0.28	0.05	0.01	0.93	0.18	0.21	0.14	0.32	0.42	**0.003**
	Corrected correlation	−0.47	**< 0.001**	0.15	0.33	0.30	0.04	−0.09	0.55	−0.04	0.04	0.02	0.92	0.19	0.22	0.14	0.32	0.45	**0.002**
Mining herbivory	Correlation	−0.09	0.09	0.05	0.05	0.03	0.04	−0.16	0.02	−0.12	0.10	0.04	0.04	0.01	0.01	0.06	0.04	0.04	0.18
	Phyl.sign	−0.10	0.49	0.14	0.34	0.36	0.13	−0.15	0.32	−0.08	0.61	0.16	0.28	−0.18	0.21	−0.15	0.28	0.26	0.07
	Corrected correlation	−0.10	0.49	0.14	0.35	0.35	0.02	−0.14	0.35	−0.08	0.61	0.18	0.24	−0.15	0.29	−0.17	0.25	0.24	0.10

*Note:* Unadjusted significance levels are shown in table (*p*) and Bonferroni‐adjusted significant results (*p* < 0.0056) are marked with in bold.

Abbreviations: C, carbon content; CN_ratio, ratio between carbon and nitrogen; CP, available crude protein; CT, percent of condensed tannins; FreshW, fresh weight of leaves; LDMC_leaf, leaf dry matter content; N, nitrogen content; P, phosphorus content; SLA, specific leaf area.

#### 2.4.1 Vegetation Type Level

Second, we tested whether there were mean differences in any of the insect herbivore types (total, chewer, miner) among the three vegetation types. We calculated community weighted mean herbivory estimates per plot, using estimates for herbivory on each species in the plot weighted by its abundance based on basal area. We employed generalised linear mixed models (GLMMs) to analyse the data, focusing on the effects of type of herbivory (chewing or mining), vegetation type (savanna, thicket or forest), and their interaction on herbivory rates. These variables were included as fixed factors, while plant species and plant individuum code was treated as a random factor to account for variability. Note that plant individuum code inherently included the nested structure of plants within species and species within plots (e.g., species_plot_id). The response variables were the three different measures of mean herbivory (total, chewing, mining), and we assumed beta error distributions appropriate for proportional data, implemented using the ‘glmmTMB’ function in the ‘glmmTMB’ package (Brooks et al. 2017). To identify the most parsimonious model, we constructed all possible subset models combining the fixed factors and compared their fit using ΔAICc (Akaike Information Criterion corrected for small sample sizes). We selected the simplest model with a ΔAICc value within 2 units of the minimum observed AICc as the best‐fit model.

Additionally, to assess herbivory patterns consistently across different habitats, we performed a subset analysis on data from seven plant species that were sampled across all plots. In this analysis, plant species was included as a fixed factor, and plant individual was treated as a random factor to control for within‐species variation. Similar GLMMs were constructed and evaluated for this subset, following the same model selection criteria.

#### 2.4.2 Plot Level

Finally, we subsetted the data for those plots collected in savannas only (24 plots) and assessed how community‐weighted rates of herbivory depended on the fire frequency in the last 20 years (number per year) and the abundance of mammals (expressed by the abundance of dung of large mammals per plot). First, as fire frequency and dung counts were highly negatively correlated (*N* = 24, *p* < 0.001, *R* = −0.639), we ran a PCA to distribute the plots along an axis of trade‐off between fire‐dominated plots and mammal herbivory‐dominated plots. After that, we obtained the PCA axis score and plotted it against the total herbivory damage, and fitted the data using nonlinear regression, with the best fit provided by a polynomial function. Then we tested whether total herbivory and herbivory by chewers or miners was related to the fire‐herbivory PCA axis or to rainfall (Table S6).

## 3 Results

**FIGURE 2:**
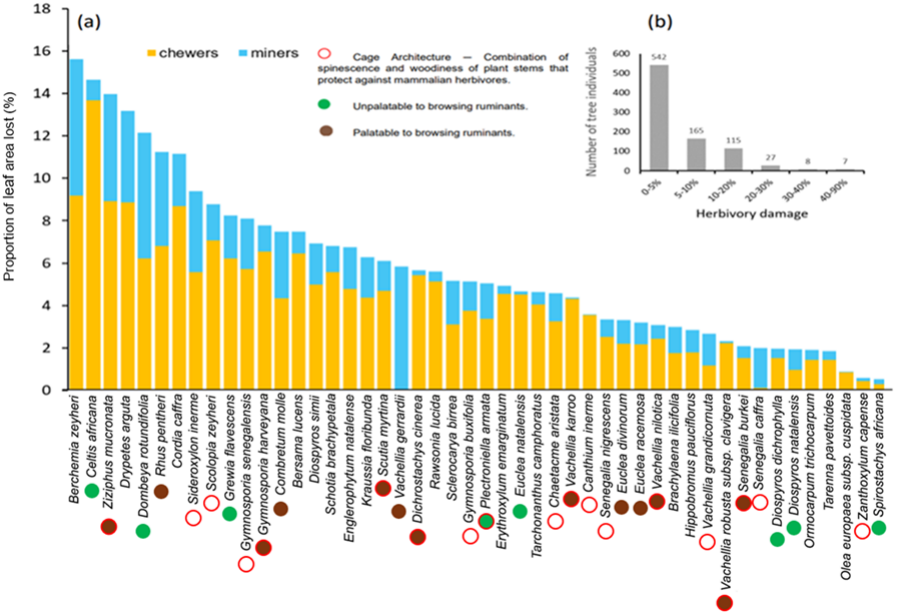
Mean proportion of leaf area eaten by chewers and miners per individual plant species, with notes on known defences against mammal herbivores of the plant species (a) and the number of tree individuals across all species suffering various levels of mean total herbivory damage (b). Plant structural defence is marked with a red circle, indicating cage architecture (thorns and branches) protecting the plant against herbivory by goats (Charles‐Dominique et al. 2017; Owen‐Smith and Cooper 1987). A green dot signifies woody species unpalatable to browsing ruminants (impalas, kudus, and goats), while a brown dot indicates woody species palatable to browsing ruminants, based on the palatability classification of Owen‐Smith and Cooper (1987) and additional information from Charles‐Dominique et al. (2017). Refer to Table S3 for plant species notes regarding structural defence and palatability. *Rhus pentheri* is a synonym of *Searsia pentheri* (See Table S2). Actual herbivory values per tree species are provided in Table S4.

In total, we surveyed herbivory damage caused by insects on 26,244 leaves from 864 woody plant individuals belonging to 48 plant species collected from 38 plots (7 in forests, 24 in savannas and 7 in thicket). There was wide variation in the amount and source of damage among individuals and tree species (ranging from 0% to 90% and 0% to 16%, respectively, Figure 2, Table S3). Most individuals suffered minor damage (< 5%), with mean damage of 2.97% ± 4.94% of leaf area. Only 42 individuals suffered insect herbivory between 20% and 50%. In general, mean damage caused by chewers was more than 2 times higher (4.20% ± 0.10%) than damage caused by miners (1.80% ± 0.10%; *Z* = 25.80, *p* = 0.001), and there was substantial variability in feeding guild‐specific leaf damage across plant species.

### 3.1 Insect Herbivory at the Level of Plant Species: Nutrient and Mammal Herbivory

We found that, across plant species, total and chewing herbivory but not mining herbivory were negatively correlated with species‐level carbon content (C), only with phylogenetic information for the tree species (Table 1). However, both chewing and mining herbivory were significantly positively correlated with nitrogen content with phylogenetic information. Meanwhile, phosphorus content (P), ratio between carbon and nitrogen (CN_ratio), percent of condensed tannins (CT) and available crude protein (CP) did not have any relationship to either types of insect herbivory or their total.

We found no trade‐off between insect herbivory and herbivory by mammals across plant species. We did not find any significant correlation between any herbivory type (chewer, miner) and either palatability ranking for mammals or structural defences. The BSI of mammals (Charles‐Dominique, Midgley, and Bond 2015) and insect herbivory of individual plant species showed no significant correlation (*r* = 0.100, *p* = 0.370 for herbivory by chewers; *r* = 0.057, *p* = 0.700 for herbivory by miners). Plant species that suffered relatively high insect herbivory included species with both low (*Berchemia zeyheri*, *Celtis africana*, *Dombeya rotundifolia*) and high structural defences (*Z. mucronata*) (Figure 2, Tables S2 and S3).

### 3.2 Insect Herbivory at the Level of Vegetation Type – Across Forests, Savannas and Thickets

**FIGURE 3:**
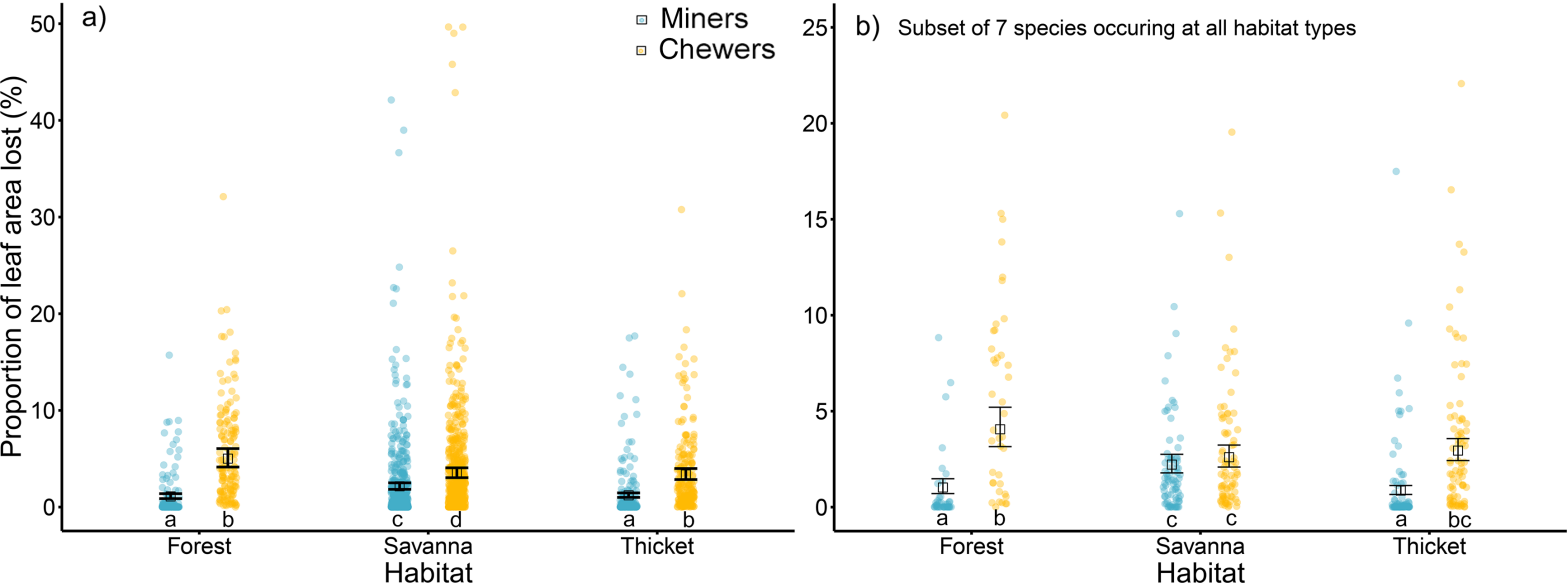
Effect of vegetation type and type of herbivory on the mean (±SE) proportion of leaf area lost for the full dataset, at the level of the vegetation type, including 48 plant species (a), and for the subset of seven plant species occurring in all study plots (b). Individual data points represent individual trees which were treated as random factor. The code for the individuals inherently included the nested structure of plants within species and species within plots. Significant differences (*p* < 0.05) between the types of herbivory in interaction with the type of vegetation are denoted by different letters just above the *x*‐axis. Note the different scales of the *y*‐axis.

The best model explaining insect herbivory damage, when considering all individuals of the 48 species, included vegetation type, type of herbivory, and the interaction between vegetation type and type of herbivory (Table 2, Table S5). Total insect herbivory (i.e., the sum of chewing and mining herbivory) was greater in forest (7.20% ± 11.30%) and thicket (7.10% ± 9.60%) than in savanna (4.20% ± 6.40%). Mean leaf area lost to chewers was significantly higher in forests (5.0% ± 0.40%) than in thickets (3.40% ± 0.30%) and savannas (3.50% ± 0.30%) (Table 2, Figure 3). Mean leaf area lost to miners was significantly higher in savannas (2.20% ± 0.10%) than in thickets (1.20% ± 0.10%) and forests (1.10% ± 0.10%) (Figure 3).

**TABLE 2:** Resulting models explaining herbivorous damage in full dataset (a) and in the dataset subset to 7 plant species occurring in all plots (b).

No.	Vegetation type	Type of herbivory	Species	Vegetation type: type of herbivory	Vegetation type: species	Species: type of herbivory	df	AICc	delta
**(a) Full dataset (species and individual was used as random factor)**									
1	+	+	NA	+	NA	NA	9	−14,979.0	0.0
2	+	+	NA	−	NA	NA	7	−14,970.3	8.9
3	−	+	NA	−	NA	NA	5	−14,961.8	17.3
**(b) Subset of 7 plant species (only individual used as random factor)**									
1	+	+	+	+	+	+	32	−2535.9	0.0
2	+	+	+	+	−	+	20	−2531.5	4.5
3	+	+	+	+	+	−	26	−2526.9	9.0
4	+	+	+	+	−	−	14	−2522.7	13.3
5	+	+	+	−	+	+	30	−2503.3	32.6
6	+	+	+	−	−	+	18	−2498.9	37.0
7	−	+	+	−	−	+	16	−2495.8	40.1
8	+	+	+	−	+	−	24	−2489.3	46.6
9	+	+	+	−	−	−	12	−2487.1	48.8
10	−	+	+	−	−	−	10	−2483.1	52.8
11	+	+	−	+	−	−	8	−2468.3	67.6
12	+	+	−	−	−	−	6	−2450.8	85.1
13	−	+	−	−	−	−	4	−2445.3	90.7
14	+	−	+	−	+	−	23	−2407.7	128.3
15	+	−	+	−	−	−	11	−2403.2	132.7
16	−	−	+	−	−	−	9	−2386.1	149.8
17	+	−	−	−	−	−	5	−2385.0	150.9
18	−	−	−	−	−	−	3	−2372.3	163.6

Abbreviations: −, variable not included in the model; +, variable included in the model; NA, variable not considered in the models.

Differences among vegetation types across only the seven species occurring in all three vegetation types were consistent with differences when we included all species (Table 2, Figure 3). Insect herbivory damage varied depending on the vegetation type (*p* < 0.001), type of herbivory (*p* < 0.001), as well as plant species (*p* < 0.001), vegetation type in interaction with the type of herbivory (*p* < 0.001), vegetation type interacting with plant species (*p* < 0.001; Figure S4), and type of herbivory interacting with plant species (*p* < 0.001; Figure S5). In summary, total herbivory was higher in forests than in savannas and thickets for many plant species (but not *Scutia myrtina*, which experienced more insect damage in savannas than in forests and thickets). In all plant species except *Spirostachys africana*, chewers contributed more to overall herbivory than miners.

### 3.3 Insect Herbivory Across Environmental Gradients in Savanna

**FIGURE 4:**
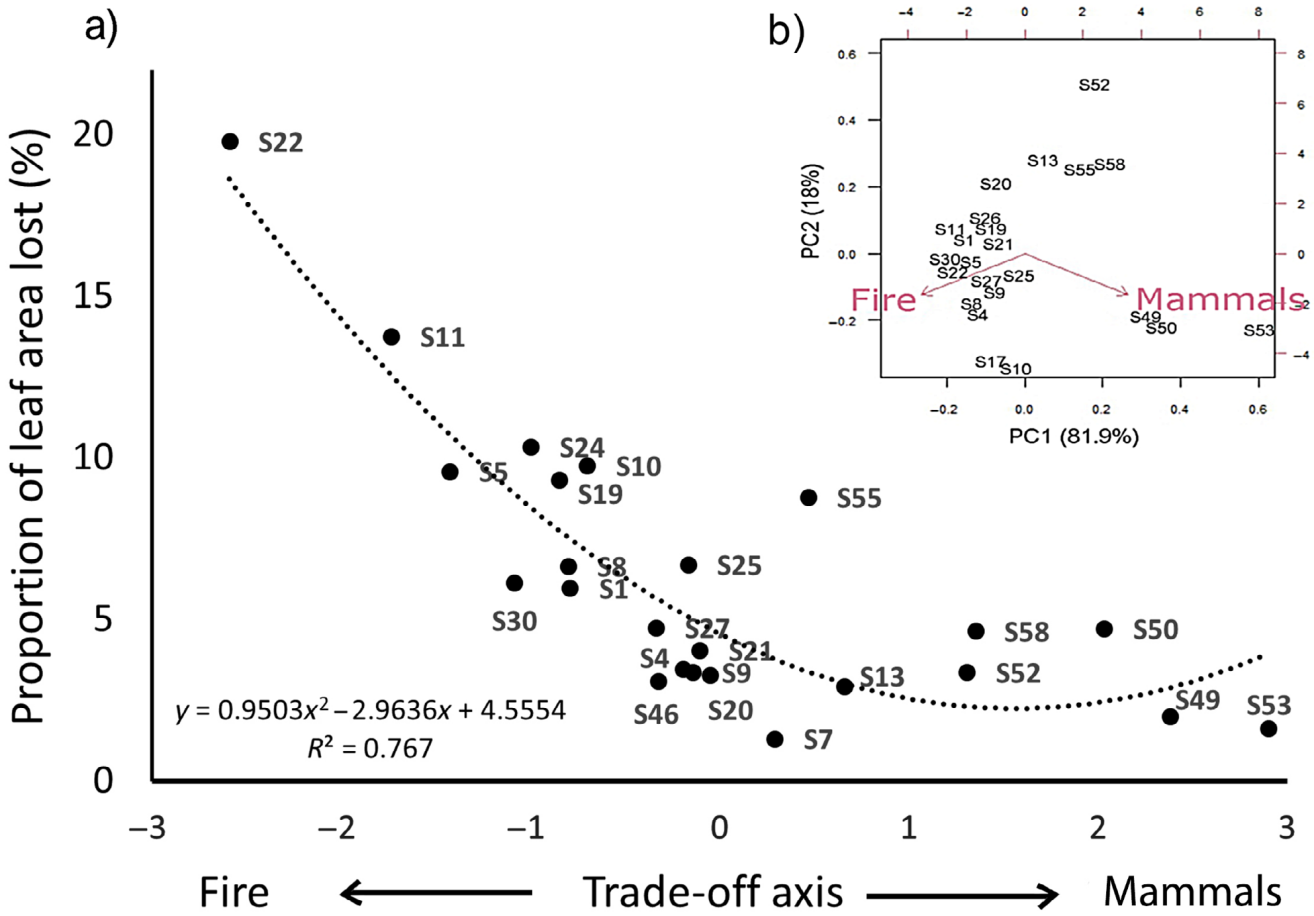
Relationship between the mean total leaf area lost to insects across savanna plots, depending on their position along a gradient of fire frequency in the past 20 years ‐mammal density (Fire‐Mammals, respectively). The data are best fitted by a polynomial function (a). PCA of the frequency of fires in the past 20 years and mammal density (represented by the number of dungs) at individual study plots (b). Data point labels correspond to Table 2, each sample represents a community‐weighted herbivory damage at a given plot (*N* = 24). Herbivory data partitioned into chewing and mining herbivory are presented in Figure S6.

Insect herbivory rates varied across savanna plots, and model selection via AICc showed that the best supported model included the fire frequency‐mammal abundance gradient and the type of insect but not rainfall (Table S6). The plots with highest fire frequency and lowest abundance of mammals had the highest herbivory by insects (Figure 4, Figure S6 for each insect herbivory type).

## 4 Discussion

To our knowledge, this study marks the first comprehensive report on insect herbivory particularly for chewers and miners in an African savanna‐forest mosaic. We found that mean insect herbivory across all vegetation types of HiP was 6%. This is comparable to global estimates for temperate forests and about half of the herbivory damage reported from tropical forests (Coley and Aide 1991) and falls just below the estimated global averages – certainly not negligible. Moreover, insect damage was not uniformly distributed, with some plant individuals suffering herbivory damage up to 80% and several plant species suffering mean herbivory damage > 10%. The impact of herbivory varied according to insect feeding guilds and vegetation types, chewers having the strongest influence overall, favouring forest while miner herbivory were more important in savanna. Plants with high C, LDMC, SLA and N were generally preferred by insect herbivores. However, P, CP, CN_ratio and CT, which are factors that typically influence mammal herbivory, had no effect on insect herbivory. This is coherent with the tendency of insects to avoid plants that are structurally defended against mammals, as well as locations with higher mammal abundance (Becerra 2015; Perkovich and Ward 2022). This suggests that plants may adapt differently depending on the type of herbivore, although no trade‐off was observed between mammal and insect preferences. Finally, insect herbivory increased with fire but was independent of rainfall. Altogether, this study provides important insights on the drivers of insect herbivory. Such levels of insect herbivory are known to accelerate nutrient cycling and increase plant production in grasslands (Belovsky and Slade 2000) and might be comparable to herbivory by mammals in some habitats, even though they differ mechanistically. Analysis of various mammal exclosures found that mammalian herbivory in African savanna ecosystems results in a 57% biomass loss for grass and a 30.6% loss for trees across Africa (Staver et al. 2021). Furthermore, mammalian herbivory differs significantly from insect herbivory because mammals, particularly terrestrial mammals, tend to uproot and trample plants and remove apical meristems when browsing. This is comparatively more challenging for plants to face damage by mammals than to replace leaf foliage caused by insects, so mammals may be having a larger impact on plant communities. Nevertheless, the loss of biomass to insect herbivores in open ecosystems (savannas, thickets) is clearly substantial.

### 4.1 Niche Partitioning of Insect Herbivore Guilds (Chewers and Miners) Linking to Plant Species and Vegetation Types

We hypothesised that woody plant species in savannas would experience less insect herbivory than forests (forest and thicket) due to greater competition for food resources with large mammals in savannas. However, the data did not support our hypothesis. High variability was observed in the type of insect herbivory (chewer and miner) in all woody plant species across the three vegetation types (forest, savanna and thicket). Insect leaf damage was dominated by chewing damage, whereas mining damage was lower across all woody plant species except *Vachellia gerrardii* and *Senegalia caffra*. Overall, chewing herbivory was relatively higher than mining herbivory, ca. 4% versus 2%, respectively. This pattern was similarly observed in other studies (Dole, Menges, and David 2023; Gossner et al. 2014; Nooten and Hughes 2013; Pearse and Hipp 2009). Moreover, we found that the highest leaf damage from miners ranged between 5% and 6%, which were observed only in four plant species, namely *Berchemia zeyheri*, *Ziziphus mucronata*, *Dombeya rotundifolia* and *Vachellia gerrardii*. We also noted that miners not only preferred plants with larger leaves but also those with smaller leaves, (e.g., *V. gerrardii*). For example, more than 94% of the foliage of *Senegalia caffra* (smaller leaves) was eaten by miners. Hence, these combined effects of herbivory types indicate that the preference of various herbivore guilds may be influenced by plant traits (Muiruri et al. 2019), or previous damage of woody plants leaves by insects and the plant community dynamics (Basset 1991; Faeth 1985; Martini and Goodale 2020).

Although chewing herbivory was higher in all three vegetation types within HiP, when considering chewers and miners separately against vegetation types, we found that mining herbivory was higher in savanna than in forest and thicket. The relatively higher damage caused by miners in savanna is a surprising finding, as miners are expected to be more frequent in less sclerophyllous plants. These results might be explained by the absence of pronounced defences of plants against herbivory from miners specifically, and by a high degree of specialisation of that insect guild (Bairstow et al. 2010) or because of plant availability and lower water content (Rossetti et al. 2014).

Furthermore, in our investigation of seven plant species that were present in all three vegetation types, we observed that rates of insect herbivory were quite similar to those observed at the community level. This suggests that the disparities observed among vegetation types were primarily influenced by variations in plant species composition, and the herbivory patterns specific to each plant species, which remained relatively consistent across all three vegetation types. Only one species, *Scutia myrtina*, suffered significantly higher herbivory in savanna than in forest and thicket.

### 4.2 Defence Mechanisms in Woody Plants Versus Herbivory Type (Mammals vs. Insects)

We had anticipated that plants adapted to high mammal herbivory through structural defences would be more poorly defended against insects, and thus suffer more severe herbivory, but our analyses did not support this hypothesis. Several species suffered high herbivory by insects and low herbivory by mammals, but many plant species seemed to be well protected against all types of herbivory (chewing and mining). For example, *Ziziphus mucronata* effectively protected its foliage against mammals with a cage of thorns and branches but lost 14% of its foliage to various insects, while white stinkwood (*Celtis africana*) was unpalatable for mammals to digest, yet it lost foliage to insects at similar rates (~15%), mostly to chewers. African sandalwood (*Spirostachys africana*) represented a species from the other end of the spectrum, completely avoided by mammals and rarely suffering from insect herbivory (0.5%), although large herbivores such as elephant and rhinoceros have been reported to feed on young foliage (Kopong and Mojeremane 2012). *Spirostachys africana* produces a toxic milky latex and its bark contains diterpenoids, triterpenoids, tannins, anthocyanins and saponins, which may possibly inhibit herbivores (Singh, Baijnath, and Street 2020; Kopong and Mojeremane 2012).

Moreover, there was evidence that plant communities subjected to higher fire frequencies are more susceptible to insect herbivory than communities where the main disturbance was mammalian herbivory (Figure 4). This pattern might be explained by several causes: first, drier sites tend to be affected more by mammalian herbivory while wetter sites have greater fire frequencies (Staver et al. 2012; Hempson, Archibald, and Bond 2015) and also exert a major constraint on insect development (Jaworski and Hilszczański 2013; Lin et al. 2021; Trisos et al. 2021); second, chemical defences are expected to be strongly influenced by the level of mammal herbivory. Little is known about the differences in chemical defences between fire‐ and mammalian herbivore‐driven ecosystems, but it has been speculated that there are differences in abundance of N‐based versus C‐based defences of structurally defended versus non‐structurally defended species (Hanley et al. 2007; Lopes and Vasconcelos 2011; Poeydebat et al. 2021; Schuldt et al. 2010; Tomlinson et al. 2016). Our analysis also ignores species diversity within the chewer and miner groups, as we measured the resulting herbivory damages; further investigation into the diversity responses of herbivorous insects across the fire‐vertebrate herbivory spectrum are needed to investigate this point.

### 4.3 Effects of Plant Traits on Insect Herbivory

Observed rates of insect herbivory significantly varied among plant species, decreasing with carbon and dry matter contents, respectively, and increasing with specific leaf area. SLA is indicative of leaf nutrition (Gonçalves et al. 2005), so this result agrees with previous findings obtained in pine plantations in North Carolina, suggesting that insects forage preferentially on more nutritious plants (Knepp et al. 2005). Since we correlated relative amounts of plant tissue removed, but not the total amount removed per species, this measure relates directly to animal preference rather than to its availability in the landscape, which can confound the results. By contrast, past work by Loranger et al. (2012) suggested that leaf nitrogen, lignin, leaf phosphorus content and LDMC (a different set of plant traits than we identified here) strongly shaped insect herbivory on grass and forb species. One possibility is that determinants of herbivory damage may differ among life forms. Another possibility is that plant traits strongly covary, and that suites of traits are more informative than individual plant traits. For instance, leaf N tends to increase with SLA, and so both may represent the same process (Wright et al. 2004). We unfortunately did not sample certain traits, particularly secondary metabolites which prevented us from providing resolution on this issue (Erb and Kliebenstein 2020).

### 4.4 Relationship of Insect Herbivory and Fire Frequency in the Savanna Vegetation

We hypothesised that insect herbivory would increase with fire frequencies within the savanna biome, because fire promotes resprouting of soft and undefended leaves, thereby accelerating nutrient cycling and supporting insect herbivory. Our data support this hypothesis, as insect herbivory was positively related to fire frequency over 20 years prior to the sampling. However, it is important to note that fire frequency is not the best factor, and the time from the last fire would be potentially much better explanatory factor. Such data were however unavailable to us. Furthermore, fire can indirectly impact herbivores by altering the quality of the plants they consume. For instance, it can increase the crude protein content in leaves (Greene, Hebblewhite, and Stephenson 2012) or influence soil properties such as soil carbon levels and water‐holding capacity (Kitzberger et al. 2005). These alterations in soil properties, in turn, can affect the nutrient content of plants (Huang and Boerner 2007). The complexity of these outcomes is further confounded by species interactions. For instance, while fire on its own might not significantly impact insect communities, but when combined with the presence of grazing mammals, it has the potential to decrease both the richness and abundance of arthropods (Bailey and Whitham 2002; Jonas and Joern 2007).

### 4.5 Relationship of Insect Herbivory and Rainfall in the Savannas

Finally, we hypothesised that there would be a positive relationship between insect herbivory and rainfall among savanna plots. However, we did not find significant correlation between rainfall and insect herbivory in savannas. This could mean that other factors, such as predation pressure, could be controlling insects' population dynamics (Weissflog et al. 2018). Similar to our result, Smith and Williams (2023) also observed complex responses of plant and herbivorous insect communities to rainfall manipulation in an oak savanna grassland, with no clear correlation between the two. Furthermore, a study conducted by Neves et al. (2010) in Neotropical forest‐savanna transition suggested that herbivory rates in this habitat are driven by soil nutrient content, rather than rainfall. Taken together, these studies could indicate that the relationship between rainfall and insect herbivory in savannas is not straightforward and may require careful consideration of plant species, nutrients uptake and predator–prey interactions across rainfall gradients.

## 5 Concluding Remarks

Our examination of insect herbivory showed that insect herbivory in savannas is far from negligible and should not be ignored, despite being lower than global mean estimates. Moreover, some species experienced much higher insect herbivory than others, which suggests that insect herbivory could have the potential to impact plant species sorting. In savannas, insect herbivory was higher in fire‐dominated systems than in large mammal‐dominated systems, and it also differed among vegetation types. While our work sheds light on the complex interactions among fire, rainfall, plants and insect herbivores, it also inspires questions for future exploration. Future studies should focus on explicitly linking plant defences to leaf damage and on surveying arthropod communities that feed on plants. Seasonality likely also deserves more explicit attention, as herbivory damage accumulates over the lifespan of the leaf, which varies especially across evergreen versus deciduous species. Finally, future work should explore the interaction between rainfall and fire regimes in more detail at the local scale, as potential changes in rainfall and fire regimes might affect insect communities and their interactions with plants in largely unknown directions (Koltz et al. 2018).

## Author Contributions

**Heveakore Maraia:** conceptualization (equal), data curation (equal), formal analysis (equal), investigation (equal), software (equal), visualization (equal), writing – original draft (lead). **Tristan Charles‐Dominique:** conceptualization (equal), investigation (equal), methodology (equal), resources (equal), validation (equal), visualization (equal), writing – review and editing (equal). **Kyle W. Tomlinson:** investigation (equal), validation (equal), visualization (equal), writing – review and editing (equal). **Ann Carla Staver:** investigation (equal), methodology (equal), resources (equal), validation (equal), visualization (equal), writing – review and editing (equal). **Leonardo Re Jorge:** investigation (equal), validation (equal), visualization (equal), writing – review and editing (equal). **Uriel Gélin:** investigation (equal), validation (equal), visualization (equal), writing – review and editing (equal). **Jitka Jancuchova‐Laskova:** data curation (equal), investigation (equal), validation (equal), visualization (equal), writing – review and editing (equal). **Legi Sam:** investigation (equal), validation (equal), visualization (equal), writing – review and editing (equal). **Dawood Hattas:** investigation (equal), resources (equal), validation (equal), visualization (equal), writing – review and editing (equal). **Inga Freiberga:** data curation (equal), investigation (equal), validation (equal), visualization (equal), writing – review and editing (equal). **Katerina Sam:** conceptualization (equal), data curation (equal), formal analysis (equal), funding acquisition (full), investigation (equal), methodology (equal), project administration (equal), resources (equal), software (equal), supervision (equal), validation (equal), visualization (equal), writing – review and editing (equal).

## Conflicts of Interest

The authors declare no conflicts of interest.

## Supporting information


Data S1

## Data Availability

The data are available from Zenodo (https://doi.org/10.5281/zenodo.10853273).
