# Supplementary material for: Substantial Insect Herbivory in a South African Savanna‐Forest Mosaic: A Neglected Topic
Source: Ecol Evol. 2024 Nov 9;14(11):e70466. doi: 10.1002/ece3.70466 (PMC11549574; doi:10.1002/ece3.70466)
Supplement: Supplementary file 1 — Data S1 [file ECE3-14-e70466-s001.docx]

**JOURNAL: ECOLOGY AND EVOLUTION**

**TITLE: Substantial insect herbivory in a South African savanna-forest mosaic: a neglected topic**

Heveakore Maraia^1,2*^, Tristan Charles-Dominique^3,4^, Kyle W. Tomlinson^7^, Ann Carla Staver^5^, Leonardo Re Jorge^1,2^, Uriel Gélin^8^, Jitka Jancuchova-Laskova^1,2^, Legi Sam^1^, Dawood Hattas^6^, Inga Freiberga^1^, & Katerina Sam^1,2^

^1^Biology Centre, Czech Academy Sciences, Institute of Entomology, Branišovská 1160/31, 370 05, České Budějovice, Czech Republic

^2^Faculty of Science, University of South Bohemia, Branišovská 1760/31A, České Budějovice 2, 370 05 České Budějovice, Czech Republic

^3^UMR AMAP, University of Montpellier, CIRAD, CNRS, INRAE, IRD, Montpellier, France

^4^CNRS UMR7618; Sorbonne University; Institute of Ecology and Environmental Sciences Paris; 4, place Jussieu 75005 PARIS

^5^Ecology and Evolutionary Biology and the Yale Institute for Biospheric Studies; 165 Prospect Street, New Haven, CT 06511-8934

^6^Department of Biological Sciences, University of Cape Town, Rondebosch, Cape Town, 7700, South Africa

^7^Center for Integrative Conservation & Key Laboratory for Conservation of Tropical Rainforests and Asian Elephants, Xishuangbanna Tropical Botanical Garden, Chinese Academy of Sciences, Mengla, Yunnan 666303, China

^8^Centre for Biodiversity Dynamics in a Changing World (BIOCHANGE) and Centre for Ecological Dynamics in a Novel Biosphere (ECONOVO), Section of EcoInformatics and Biodiversity, Department of Biology, Aarhus University, Aarhus, Denmark

^*^Corresponding author: maraiah205@gmail.com

**SUPPORTING INFORMATION**

TABLE S1. The codes for vegetation type and the number of fires that occurred over a period of 20 years respectively between 1992-2012, within the studied plots, including the mean annual rainfall (MAR), soil type and dung count. Data for fire frequencies were extracted from Hluhluwe iMfolozi Park records adapted by Charles‐Dominique et al. (2015) while MAR data was derived from a rainfall map provided by the dung beetle research station

| Codes | Vegetation type | 20yr_fire | Avg_Rain | SoilType | Dung count |
| --- | --- | --- | --- | --- | --- |
| F1 | Forest | NA | 825 | Sandstone | 6 |
| F2 | Forest | NA | 825 | Sandstone | 1 |
| F3 | Forest | NA | 825 | Sandstone | 3 |
| F4 | Forest | NA | 825 | Basalt | 7 |
| F5 | Forest | NA | 825 | Sandstone | 1 |
| F6 | Forest | NA | 825 | Sandstone | 0 |
| F7 | Forest | NA | 825 | Sandstone | 8 |
| S1 | Savanna | 11 | 675 | Sandstone | 3 |
| S4 | Savanna | 12 | 675 | Sandstone | 14 |
| S5 | Savanna | 12 | 675 | Sandstone | 3 |
| S7 | Savanna | 12 | 825 | Sandstone | 27 |
| S8 | Savanna | 12 | 825 | Sandstone | 12 |
| S9 | Savanna | 11 | 825 | Sandstone | 14 |
| S10 | Savanna | 12 | 825 | Basalt | 27 |
| S11 | Savanna | 11 | 825 | Sandstone | 2 |
| S13 | Savanna | 7 | 775 | Sandstone | 11 |
| S19 | Savanna | 10 | 775 | Sandstone | 7 |
| S20 | Savanna | 9 | 775 | Mixture of silt, sand, clay and gravel | 3 |
| S21 | Savanna | 10 | 775 | Sandstone | 11 |
| S22 | Savanna | 12 | 775 | Basalt | 4 |
| S24 | Savanna | 10 | 775 | Basalt | 6 |
| S25 | Savanna | 10 | 775 | Basalt | 19 |
| S27 | Savanna | 11 | 775 | Basalt | 13 |
| S30 | Savanna | 12 | 775 | Basalt | 1 |
| S46 | Savanna | 10 | 725 | Sandstone | 6 |
| S49 | Savanna | 6 | 725 | Sandstone | 56 |
| S50 | Savanna | 6 | 725 | Basalt | 55 |
| S52 | Savanna | 4 | 725 | Sandstone | 13 |
| S53 | Savanna | 3 | 725 | Sandstone | 80 |
| S55 | Savanna | 6 | 725 | Basalt | 20 |
| S58 | Savanna | 5 | 725 | Sandstone | 26 |
| T9 | Thicket | 0 | 775 | Sandstone | 23 |
| T12 | Thicket | 0 | 775 | Sandstone | 30 |
| T13 | Thicket | 0 | 675 | Basalt | 19 |
| T17 | Thicket | 0 | 675 | Basalt | 23 |
| T22 | Thicket | 0 | 775 | Sandstone | 10 |
| T23 | Thicket | 0 | 775 | Sandstone | 1 |
| T24 | Thicket | 0 | 775 | Basalt | 13 |

**TABLE S2.** Scientific names of woody plant species, including the names of authors who described the species, with species having simple or compound leaves and with and without spines

| **No.** | **Family** | **Accepted Scientific Name** | **Authority** | **Type of leaf (Simple/Compound)** | **Spinescences (Present/Absent)** |
| --- | --- | --- | --- | --- | --- |
| 1 | Rhamnaceae | *Berchemia zeyheri* | (Sond.) Grubov | Simple | Absent |
| 2 | Francoaceae | *Bersama lucens* | (Hochst.) Szyszyl. | Compound (pinnate) | Absent |
| 3 | Asteraceae | *Brachylaena ilicifolia* | (Lam.) E.Phillips & Schweick. | Simple | Absent |
| 4 | Rubiaceae | *Canthium inerme* | (L.f.) Kuntze | Simple | Present |
| 5 | Cannabaceae | *Celtis africana* | Burm.fil. | Simple | Absent |
| 6 | Cannabaceae | *Chaetacme aristata* | Planch. | Simple | Present |
| 7 | Combretaceae | *Combretum molle* | R.Br. ex G.Don | Simple | Absent |
| 8 | Boraginaceae | *Cordia caffra* | Sond. | Simple | Absent |
| 9 | Fabaceae | *Dichrostachys cinerea* | (L.) Wight & Arn. | Compound (bipinnate) | Present |
| 10 | Ebenaceae | *Diospyros dichrophylla* | (Gand.) De Winter | Simple | Absent |
| 11 | Ebenaceae | *Diospyros natalensis* | (Harv.) Brenan | Simple | Absent |
| 12 | Ebenaceae | *Diospyros simii* | (Kuntze) De Winter | Simple | Absent |
| 13 | Malvaceae | *Dombeya rotundifolia* | (Hochst.) Planch. | Simple | Absent |
| 14 | Putranjivaceae | *Drypetes arguta* | (Müll.Arg.) Hutch. | Simple | Absent |
| 15 | Sapotaceae | *Englerophytum natalense* | (Sond.) T.D.Penn. | Simple | Absent |
| 16 | Erythroxylaceae | *Erythroxylum emarginatum* | Thonn. | Simple | Absent |
| 17 | Ebenaceae | *Euclea divinorum* | Hiern | Simple | Absent |
| 18 | Ebenaceae | *Euclea natalensis* | A.DC. | Simple | Absent |
| 19 | Ebenaceae | *Euclea racemosa* | L. | Simple | Absent |
| 20 | Malvaceae | *Grewia flavescens* | Juss. | Simple | Absent |
| 21 | Celastraceae | *Gymnosporia buxifolia* | (L.) Szyszyl. | Simple | Present |
| 22 | Celastraceae | *Gymnosporia harveyana* | Loes. | Simple | Present |
| 23 | Celastraceae | *Gymnosporia senegalensis* | (Lam.) Loes. | Simple | Present |
| 24 | Sapindaceae | *Hippobromus pauciflorus* | (L.fil.) Radlk. | Simple | Absent |
| 25 | Rubiaceae | *Kraussia floribunda* | Harv. | Simple | Absent |
| 26 | Oleaceae | *Olea europaea* subsp*. cuspidata* | (L.) (Wall. & G.Don) Cif. | Simple | Absent |
| 27 | Fabaceae | *Ormocarpum trichocarpum* | (Taub.) Engl. | Compound (imparipinnate) | Absent |
| 28 | Rubiaceae | *Plectroniella armata* | (K.Schum.) Robyns | Simple | Present |

| **TABLE S2.** Continued | | | | | |
| --- | --- | --- | --- | --- | --- |
|  | | | | | |
| **No.** | **Family** | **Accepted scientific name** | **Authority** | **Type of leaf (Simple/Compound)** | **Spinescence (Present/Absent)** |
| 29 | Achariaceae | *Rawsonia lucida* | Harv. & Sond. | Simple | Absent |
| 30 | Fabaceae | *Schotia brachypetala* | Sond. | Compound (pinnate) | Absent |
| 31 | Anacardiaceae | *Sclerocarya birrea* | (A.Rich.) Hochst. | Compound (pinnate) | Absent |
| 32 | Salicaceae | *Scolopia zeyheri* | (Nees) Szyszyl. | Simple | Present |
| 33 | Rhamnaceae | *Scutia myrtina* | (Burm.fil.) Kurz | Simple | Present |
| 34 | Anacardiaceae | *Searsia pentheri* | (Zahlbr.) Moffett | Simple | Absent |
| 35 | Fabaceae | *Senegalia burkei* | (Benth.) Kyal. & Boatwr. | Compound (bipinnate) | Present |
| 36 | Fabaceae | *Senegalia caffra* | (Thunb.) P.J.H.Hurter & Mabb. | Compound (bipinnate) | Present |
| 37 | Fabaceae | *Senegalia nigrescens* | (Oliv.) P.J.H.Hurter | Compound (bipinnate) | Present |
| 38 | Sapotaceae | *Sideroxylon inerme* | (L.) | Simple | Absent |
| 39 | Euphorbiaceae | *Spirostachys africana* | Sond. | Simple | Absent |
| 40 | Asteraceae | *Tarchonanthus camphoratus* | (L.) | Simple | Absent |
| 41 | Rubiaceae | *Tarenna pavettoides* | (Harv.) Sim | Simple | Absent |
| 42 | Fabaceae | *Vachellia gerrardii* | (Benth.) P.J.H.Hurter | Compound (bipinnate) | Present |
| 43 | Fabaceae | *Vachellia grandicornuta* | (Gerstner) Seigler & Ebinger | Compound (bipinnate) | Present |
| 44 | Fabaceae | *Vachellia karroo* | (Hayne) Banfi & Galasso | Compound (bipinnate) | Present |
| 45 | Fabaceae | *Vachellia nilotica* | (L.) P.J.H.Hurter & Mabb. | Compound (bipinnate) | Present |
| 46 | Fabaceae | *Vachellia robusta* subsp. *clavigera* | (Burch.) Kyal. & Boatwr. | Compound (bipinnate) | Present |
| 47 | Rutaceae | *Zanthoxylum capense* | (Thunb.) Harv. | Compound (pinnate) | Present |
| 48 | Rhamnaceae | *Ziziphus mucronata* | Willd. | Simple | Present |


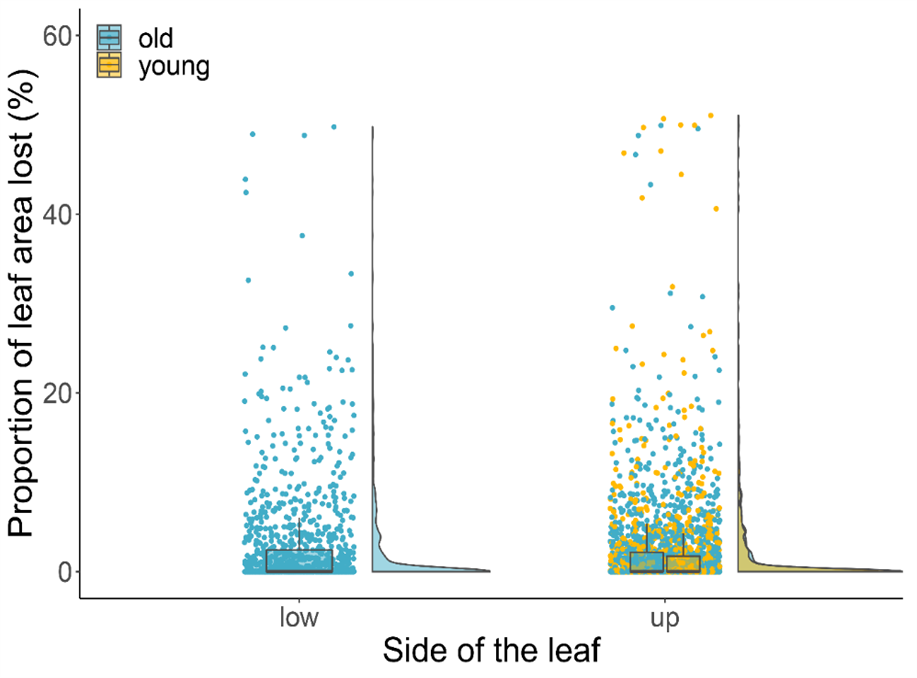


**FIGURE S1**. Proportion of leaf area lost on abaxial surface (low) of old leaves and adaxial surfaces (up) of old and young leaves.


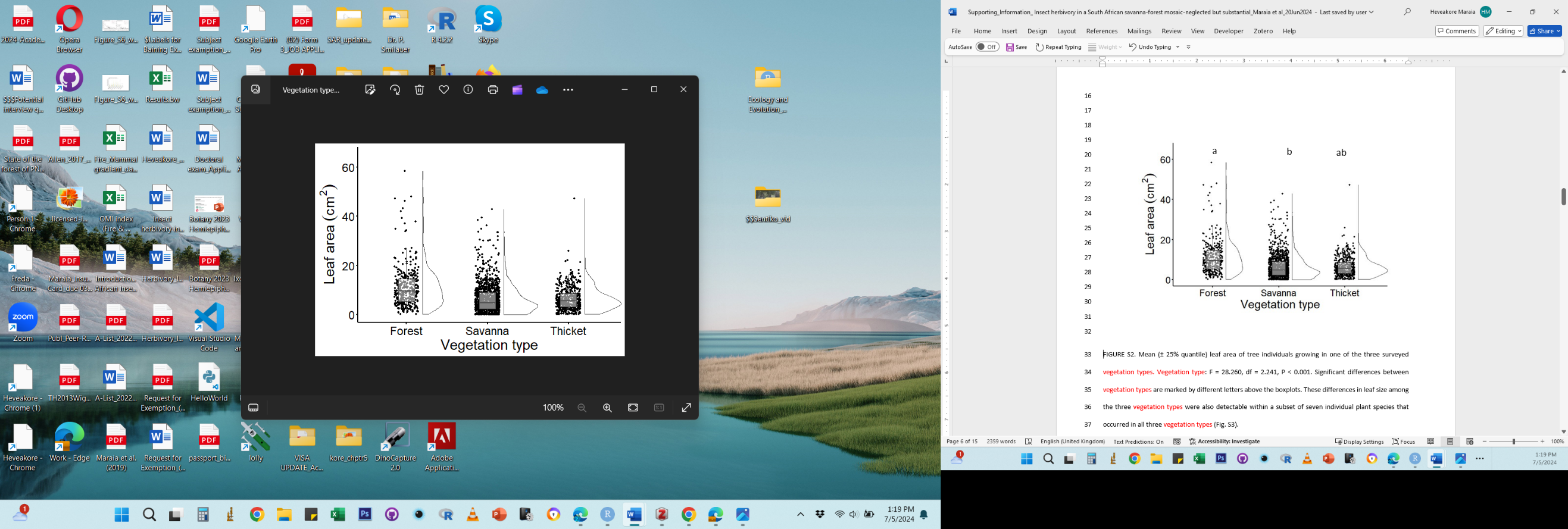


**FIGURE S2**. Mean (± 25% quantile) leaf area of tree individuals growing in one of the three surveyed vegetation types. Vegetation type: F = 28.260, df = 2.241, P < 0.001. Significant differences between vegetation types are marked by different letters above the boxplots. These differences in leaf size among the three vegetation types were also detectable within a subset of seven individual plant species that occurred in all three vegetation types (Fig. S3).


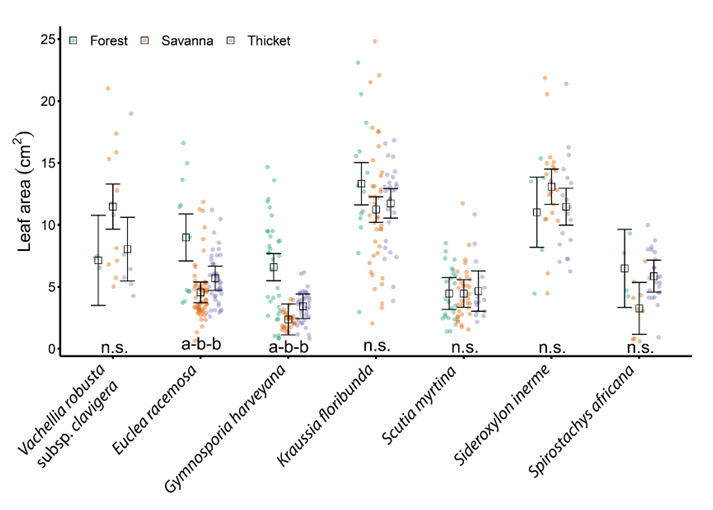


**FIGURE S3**. Mean leaf area (±SE) of plant individuals growing in different vegetation types. The graph shows a subset of 7 species that were found in all three vegetation. Significant differences between vegetation types within plant species are marked by different letters above the names of the species.

**TABLE S3**. Indicates the palatability of tree species and their structural defences (spinescence) against mammal herbivory, based on Charles‐Dominique et al. (2017) and Owen-Smith & Cooper (1987).

| Accepted Scientific name | Palatability  (Goat, kudus, impalas) | Spinescence  (Present/Absent) | Reference |
| --- | --- | --- | --- |
| *Celtis africana* | Unpalatable | Absent | **†**Charles-Dominique et al (2017) |
| *Combretum molle* | Palatable | Absent | **‡**Owen-Smith & Cooper, (1987) |
| *Dichrostachys cinerea* | Palatable | Present | **†**Charles-Dominique et al (2017); ‡Owen-Smith & Cooper, (1987) |
| *Diospyros dicrophylla* | Unpalatable | Absent | **†**Charles-Dominique et al (2017) |
| *Diospyros natalensis* | Unpalatable | Absent | **†**Charles-Dominique et al (2017) |
| *Dombeya rotundifolia* | Unpalatable | Absent | **‡**Owen-Smith & Cooper, (1987) |
| *Euclea divinorum* | Palatable | Absent | **†**Charles-Dominique et al (2017) |
| *Euclea natalensis* | Unpalatable | Absent | **‡**Owen-Smith & Cooper, (1987) |
| *Euclea racemosa* | Palatable | Absent | †Charles-Dominique et al (2017) |
| **Grewia flavescens* | Unpalatable | Absent | **†**Charles-Dominique et al (2017); ‡Owen-Smith & Cooper, (1987) |
| *Gymnosporia harveyana* | Palatable | Present | **†**Charles-Dominique et al (2017) |
| *Plectroniella armata* | Unpalatable | Present | **†**Charles-Dominique et al (2017) |
| *Searsia pentheri* | Palatable | Absent | **†**Charles-Dominique et al (2017) |
| *Scutia myrtina* | Palatable | Absent | **†**Charles-Dominique et al (2017) |
| *Senegalia burkei* | Palatable | Present | **‡**Owen-Smith & Cooper, (1987) |
| *Spirostachys africana* | Unpalatable | Absent | **†**Charles-Dominique et al (2017) |
| *Vachellia karroo* | Palatable | Present | **‡**Owen-Smith & Cooper, (1987) |
| *Vachellia nilotica* | Palatable | Present | **†**Charles-Dominique et al (2017); ‡Owen-Smith & Cooper, (1987) |
| *Vachellia robusta* subsp. *clavigera* | Palatable | Present | **†**Charles-Dominique et al (2017) |
| *Ziziphus mucronata* | Palatable | Present | **†**Charles-Dominique et al (2017); ‡Owen-Smith & Cooper, (1987) |

***** In the study by Owen-Smith & Cooper (1987), *Grewia flavescens* was found to be palatable to ruminants. However, in Charles-Dominique et al.'s (2017) study, *G. flavescens* was observed to be unpalatable. Since our study was conducted at the same site where Charles-Dominique et al. (2017) conducted their experiment, we categorised *G. flavescens* as *‘unpalatable’*.

**†** In the article entitled: The architectural design of trees protects them against large herbivores

**‡** In the article entitled: Palatability of Woody Plants to Browsing Ruminants in a South African Savanna

**TABLE S4**. Codes and names of the species and standardised mean percentage of the leaf area eaten (mean per randomly selected leaf) by chewers and miners in the three studied vegetation types (forest, savanna, and thicket).

| **No.** | **Code** | **Accepted scientific name** | **Forest** | | **Savanna** | | **Thicket** | |
| --- | --- | --- | --- | --- | --- | --- | --- | --- |
|  |  |  | **miners** | **chewers** | **miners** | **chewers** | **miners** | **chewers** |
| 1 | BER_ZEY | *Berchemia zeyheri* |  |  | 7.70 | 6.04 | 7.39 | 2.65 |
| 2 | BER_LUC | *Bersama lucens* | 6.69 | 1.07 |  |  |  |  |
| 3 | BRA_ILL | *Brachylaena ilicifolia* |  |  | 2.86 | 2.76 | 1.22 | 0.49 |
| 4 | CAN_INE | *Canthium inerme* | 3.62 | 0.05 |  |  |  |  |
| 5 | CEL_AFR | *Celtis africana* | 11.74 | 1.16 |  |  |  |  |
| 6 | CHA_ARI | *Chaetacme aristata* | 3.28 | 1.45 |  |  |  |  |
| 7 | COM_MOL | *Combretum molle* |  |  | 4.19 | 2.98 |  |  |
| 8 | COR_CAF | *Cordia caffra* |  |  | 9.36 | 2.95 | 2.75 | 0.00 |
| 9 | DIC_CIN | *Dichrostachys cinerea* |  |  | 1.81 | 0.31 | 3.34 | 1.17 |
| 10 | DIO_DIC | *Diospyros dichrophylla* |  |  | 1.43 | 0.61 | 1.75 | 0.03 |
| 11 | DIO_NAT | *Diospyros natalensis* |  |  |  |  | 1.09 | 1.51 |
| 12 | DIO_SIM | *Diospyros simii* |  |  |  |  | 5.41 | 2.17 |
| 13 | DOM_ROT | *Dombeya rotundifolia* |  |  | 6.29 | 4.36 |  |  |
| 14 | DRY_ARG | *Drypetes arguta* | 8.80 | 4.39 |  |  |  |  |
| 15 | ENG_NAT | *Ehretia rigida* | 4.87 | 1.96 |  |  |  |  |
| 16 | ERY_EMA | *Erythroxylum emarginatum* | 4.81 | 0.40 |  |  |  |  |
| 17 | EUC_DIV | *Euclea divinorum* |  |  | 2.33 | 1.03 | 2.19 | 1.40 |
| 18 | EUC_NAT | *Euclea natalensis* | 4.92 | 0.14 |  |  | 1.17 | 0.11 |
| 19 | EUC_RAC | *Euclea racemosa* | 1.80 | 0.14 | 1.81 | 1.56 | 2.85 | 0.84 |
| 20 | GRE_FLA | *Grewia flavescens* |  |  | 13.46 | 7.32 | 4.01 | 0.20 |
| 21 | GYM_BUX | *Gymnosporia buxifolia* |  |  | 3.48 | 1.42 | 6.01 | 0.21 |
| 22 | GYM_HAR | *Gymnosporia harveyana* | 8.29 | 1.12 | 3.62 | 1.56 | 5.21 | 1.46 |
| 23 | GYM_MAR | *Gymnosporia maranguensis* |  |  | 4.49 | 2.60 | 2.91 | 0.00 |
| 24 | HIP_PAU | *Hippobromus pauciflorus* |  |  | 2.220 | 1.18 | 0.86 | 0.90 |
| 25 | KRA_FLO | *Kraussia floribunda* | 7.10 | 0.32 | 3.60 | 3.58 | 3.97 | 0.50 |
| 26 | OLE_EUR | *Olea europaea* subsp*. cuspidata* |  |  |  |  | 0.89 | 0.04 |
| 27 | ORM_TRI | *Ormocarpum trichocarpum* |  |  | 1.18 | 0.48 |  |  |
| 28 | PLE_ARM | *Plectroniella armata* |  |  | 4.26 | 1.66 | 3.19 | 1.34 |
| 29 | RAW_LUC | *Rawsonia lucida* | 5.58 | 0.45 |  |  |  |  |
| 30 | SCH_BRA | *Schotia brachypetala* |  |  | 8.65 | 0.80 | 4.77 | 1.41 |
| 31 | SCL_BIR | *Sclerocarya birrea* |  |  | 2.98 | 2.13 |  |  |
| 32 | SCO_ZEY | *Scolopia zeyheri* | 7.05 | 1.45 |  |  |  |  |

| **TABLE S4**. Continued | | | | | | | | |
| --- | --- | --- | --- | --- | --- | --- | --- | --- |
| **No.** | **Code** | **Accepted scientific name** | **Forest** | | **Savanna** | | **Thicket** | |
|  |  |  | **miners** | **chewers** | **miners** | **chewers** | **miners** | **chewers** |
| 33 | SCU_MYR | *Scutia myrtina* | 4.68 | 0.02 | 5.83 | 3.66 | 3.12 | 0.10 |
| 34 | RHU_PEN | *Searsia pentheri* |  |  | 7.32 | 5.91 |  |  |
| 35 | SEN_BUR | *Senegalia burkei* |  |  | 1.39 | 0.00 | 1.52 | 1.06 |
| 36 | SEN_CAF | *Senegalia caffra* |  |  | 0.51 | 3.82 |  |  |
| 37 | SEN_NIG | *Senegalia nigrescens* |  |  | 2.05 | 0.79 |  |  |
| 38 | SID_INE | *Sideroxylon inerme* | 10.89 | 5.95 | 2.89 | 2.80 | 7.70 | 3.98 |
| 39 | SPI_AFR | *Spirostachys africana* | 2.76 | 1.28 | 0.28 | 1.16 | 0.24 | 0.15 |
| 40 | TAR_CAM | *Tarchonanthus camphoratus* |  |  | 6.88 | 2.31 | 2.43 | 0.31 |
| 41 | TAR_PAV | *Tarenna pavetoides* | 1.59 | 0.34 |  |  |  |  |
| 42 | VAC_GER | *Vachellia gerrardii* |  |  | 0.00 | 6.60 |  |  |
| 43 | VAC_GRA | *Vachellia grandicornuta* |  |  | 0.67 | 1.35 |  |  |
| 44 | VAC_KAR | *Vachellia karroo* |  |  | 3.62 | 0.19 | 0.06 | 0.36 |
| 45 | VAC_NIL | *Vachellia nilotica* |  |  | 2.34 | 0.11 | 1.39 | 0.99 |
| 46 | VAC_ROB | *Vachellia robusta* subsp. *clavigera* | 0.11 | 0.25 | 2.01 | 0.21 | 0.93 | 0.61 |
| 47 | ZAN_CAP | *Zanthoxylum capense* |  |  | 0.48 | 0.44 | 0.23 | 0.33 |
| 48 | ZIZ_MUC | *Ziziphus mucronata* |  |  | 8.71 | 4.73 | 7.76 | 3.25 |

TABLE S5. Model estimates and informal significance of the factors considered in the most parsimonious models (refer to Table 2) explaining herbivory damage in the three studied vegetation types across all 48 woody plant species (a) and across subset of seven woody plant species (b) that occurred in the three vegetation types.

| Full dataset (species and individual tree used as random factor) | Estimate | Std. Error | z value | P |
| --- | --- | --- | --- | --- |
| (Intercept -i.e., Forest, Mining herbivory) | -2.9 | 0.09 | -33.3 | <0.001 |
| habitat: Savanna | -0.5 | 0.09 | -4.8 | <0.001 |
| habitat: Thicket | -0.5 | 0.1 | -4.6 | <0.001 |
| herbivory_type: Mining | -2.02 | 0.1 | -19.0 | <0.001 |
| habitat: Savanna * herbivory_type: Mining | 0.38 | 0.1 | 3.2 | 0.001 |
| habitat: Thicket * herbivory_type: Mining | 0.44 | 0.1 | 3.3 | 0.001 |
| Subset of 7 plant species (individual used as random factor) | Estimate | Std. Error | z value | P |
| (Intercept – i.e., Forest, Mining herbivory, VAC_ROB) | -5.8 | 0.6 | -9.7 | <0.001 |
| herbivory_type: Chewing | 1.4 | 0.4 | 3.5 | <0.001 |
| habitat: Savanna | 1.2 | 0.6 | 2.0 | 0.0 |
| habitat: Thicket | 0.8 | 0.6 | 1.4 | 0.2 |
| species: EUC_RAC | 0.9 | 0.6 | 1.5 | 0.1 |
| species: GYM_HAR | 1.2 | 0.6 | 2.0 | 0.0 |
| species: KRA_FLO | 1.2 | 0.6 | 1.9 | 0.1 |
| species: SCU_MYR | 1.0 | 0.6 | 1.6 | 0.1 |
| species: SID_INE | 2.6 | 0.7 | 3.8 | <0.001 |
| species: SPI_AFR | 1.7 | 0.7 | 2.4 | 0.0 |
| herbivory_type: Chewing * species: EUC_RAC | -0.2 | 0.4 | -0.6 | 0.6 |
| herbivory_type: Chewing * species: GYM_HAR | 0.6 | 0.4 | 1.6 | 0.1 |
| herbivory_type: Chewing * species: KRA_FLO | 0.1 | 0.4 | 0.3 | 0.8 |
| herbivory_type: Chewing * species: SCU_MYR | 0.3 | 0.4 | 0.8 | 0.4 |
| herbivory_type: Chewing * species: SID_INE | 0.0 | 0.4 | 0.0 | 1.0 |
| herbivory_type: Chewing * species: SPI_AFR | -0.9 | 0.5 | -2.0 | 0.0 |
| habitat: Savanna * species: EUC_RAC | 0.1 | 0.6 | 0.1 | 0.9 |
| habitat: Thicket * species: EUC_RAC | -0.6 | 0.6 | -0.9 | 0.4 |
| habitat: Savanna *species: GYM_HAR | -0.6 | 0.6 | -1.1 | 0.3 |
| habitat: Thicket * species: GYM_HAR | -0.9 | 0.6 | -1.5 | 0.1 |
| habitat: Savanna *species: KRA_FLO | -0.2 | 0.6 | -0.4 | 0.7 |
| habitat: Thicket * species: KRA_FLO | -1.0 | 0.6 | -1.6 | 0.1 |
| habitat: Savanna *species: SCU_MYR | 0.5 | 0.6 | 0.8 | 0.4 |
| habitat: Thicket * species: SCU_MYR | -0.9 | 0.6 | -1.4 | 0.2 |
| habitat: Savanna *species: SID_INE | -1.4 | 0.7 | -2.2 | 0.0 |
| habitat: Thicket * species: SID_INE | -1.8 | 0.7 | -2.8 | 0.0 |
| habitat: Savanna *species: SPI_AFR | -0.82 | 0.8 | -1.1 | 0.3 |
| habitat: Thicket * species: SPI_AFR | -1.7 | 0.7 | -2.4 | 0.0 |
| herbivory_type: Chewing * habitat: Thicket | -1.2 | 0.2 | -5.2 | <0.001 |
| herbivory_type: Chewing * habitat: Savanna | -0.2 | 0.2 | -0.7 | 0.5 |

**FIGURE S4**. Mean proportion (± SE) of herbivory for 7 plant species that occurred in all three vegetation types. Significant differences (P < 0.05) between plant species within three vegetation types are indicated by different coloured letters below the data. Significant differences (P < 0.05) between vegetations within plant species are indicated above the data in black letters. Non-significant results are
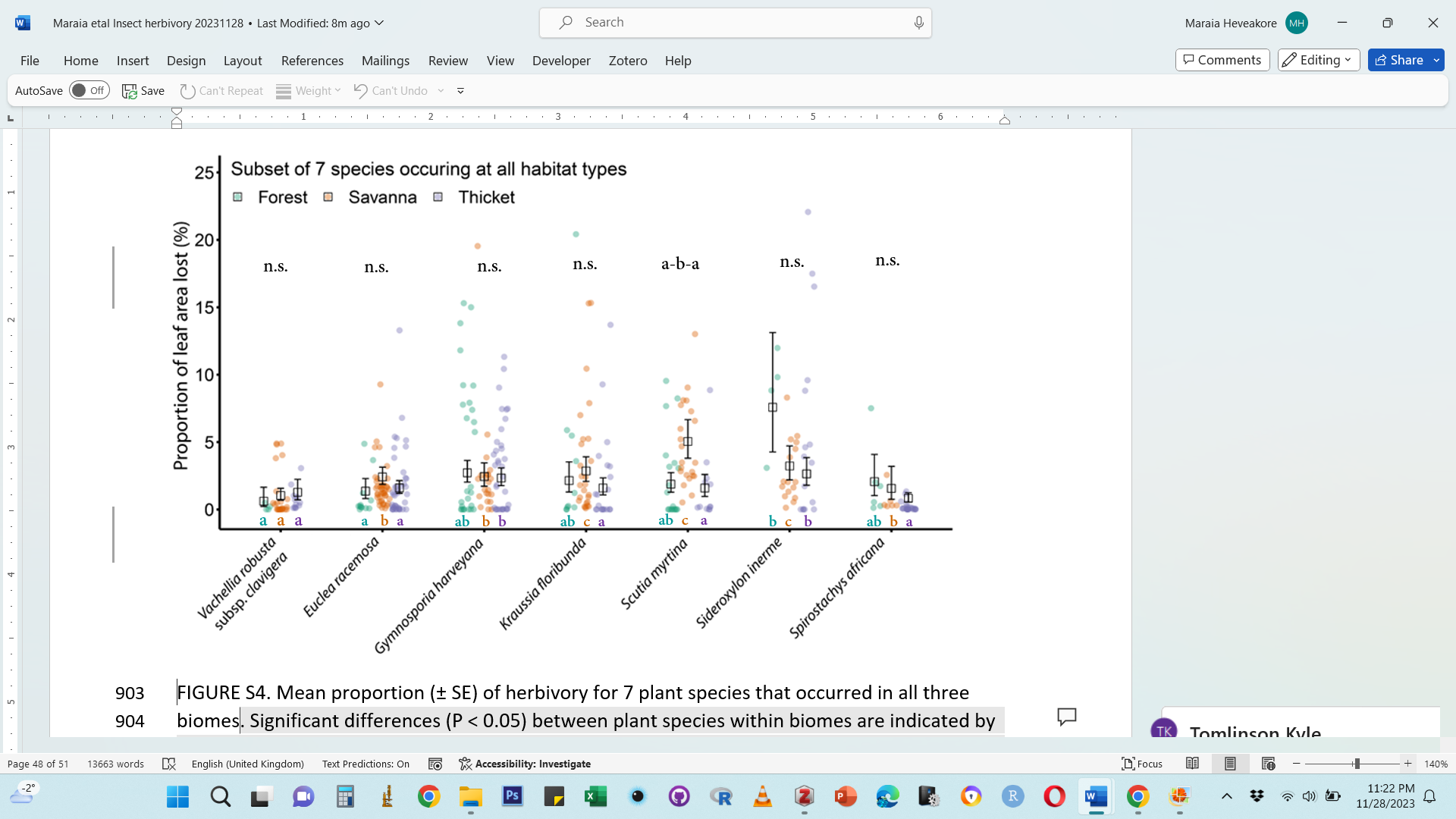
marked as n.s.


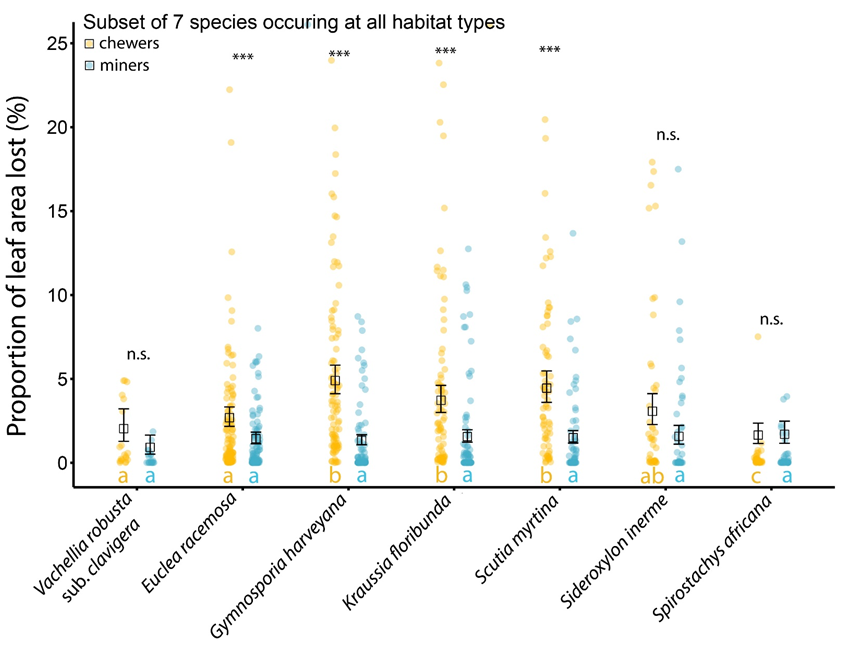


**FIGURE S5**. Proportion of leaf area lost from chewers and miners in 7 plant species that occurred in all three vegetations. Significant differences (P < 0.05) between herbivory type are indicated below the data in coloured letters. n.s. indicates non-significant between herbivory type on individual plant species. Asterisks indicate significant difference between chewers and miners on individual plant species.

**
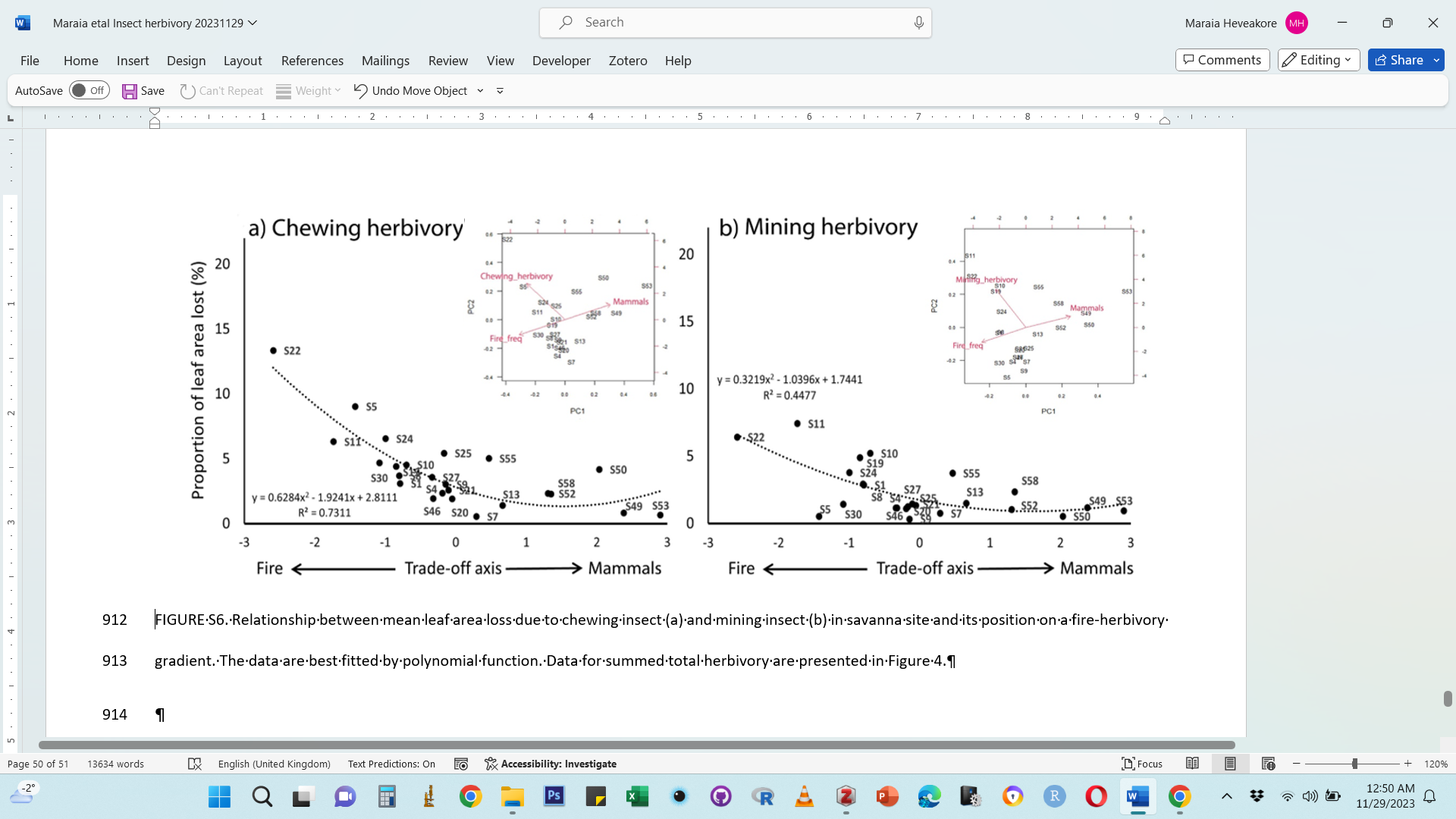
FIGURE S6**. Relationship between mean leaf area loss due to chewing insects (a) and mining insects (b) in savanna plots and their position on a fire-herbivory gradient. The data are best fitted by polynomial function. Data for summed total herbivory are presented in Figure 4. Relationship of frequency of fires in the last 20 years and mammal density (represented by the number of dungs) per plot plotted based on eigen values (from PCA) against the herbivory levels (chewing and mining herbivory separately) at individual study plots.

TABLE S6. Comparisons of multi-predictor models analysing factors (i.e., fire frequency and abundance of mammals represented by their PCA scores, rainfall, herbivory type (chewing and mining)) within the studied plots that affect the levels of herbivory. Results of the analysis of deviance based on the ΔAICc (Corrected Akaike Information Criterion). The most parsimonious models are indicated in bold for herbivory partitioned into types (a) and for total herbivory (b).

| **Herbivory (partitioned to types)** | **df** | **ΔAICc** |
| --- | --- | --- |
| **herbivory_type + PCA** | **4** | **0.0** |
| PCA + rainfall + herbivory_type | 5 | 2.4 |
| herbivory_type : PCA | 4 | 7.3 |
| PCA : rainfall | 3 | 9.0 |
| PCA | 3 | 10.8 |
| PCA + rainfall | 4 | 13.0 |
| herbivory_type | 3 | 25.0 |
| null | 2 | 28.4 |
| rainfall | 3 | 29.6 |
| **Total herbivory** | **df** | **ΔAICc** |
| **PCA ( fire_frequency - mammal_abundance)** | **3** | **0.0** |
| PCA : rainfall | 3 | 1.7 |
| PCA + rainfall | 4 | 4.6 |
| null | 2 | 21.7 |
| rainfall | 3 | 23.9 |
